# Prolonged Cerebral Circulation Time Is the Best Parameter for Predicting Vasospasm during Initial CT Perfusion in Subarachnoid Hemorrhagic Patients

**DOI:** 10.1371/journal.pone.0151772

**Published:** 2016-03-17

**Authors:** Chun Fu Lin, Sanford P. C. Hsu, Chung Jung Lin, Wan Yuo Guo, Chih Hsiang Liao, Wei Fa Chu, Sheng Che Hung, Yang Shin Shih, Yen Tzu Lin

**Affiliations:** 1 Department of Neurosurgery, Neurological Institute, Taipei Veterans General Hospital, Taipei, Taiwan; 2 Department of Radiology, Taipei Veterans General Hospital, Taipei, Taiwan; 3 School of Medicine, National Yang-Ming University, Taipei, Taiwan; Universita degli Studi di Palermo, ITALY

## Abstract

**Purpose:**

We sought to imitate angiographic cerebral circulation time (CCT) and create a similar index from baseline CT perfusion (CTP) to better predict vasospasm in patients with subarachnoid hemorrhage (SAH).

**Methods:**

Forty-one SAH patients with available DSA and CTP were retrospectively included. The vasospasm group was comprised of patients with deterioration in conscious functioning and newly developed luminal narrowing; remaining cases were classified as the control group. The angiography CCT (XA-CCT) was defined as the difference in TTP (time to peak) between the selected arterial ROIs and the superior sagittal sinus (SSS). Four arterial ROIs were selected to generate four corresponding XA-CCTs: the right and left anterior cerebral arteries (XA-CCT_RA2_ and XA-CCT_LA2_) and right- and left-middle cerebral arteries (XA-CCT_RM2_ and XA-CCT_LM2_). The CCTs from CTP (CT-CCT) were defined as the differences in TTP from the corresponding arterial ROIs and the SSS. Correlations of the different CCTs were calculated and diagnostic accuracy in predicting vasospasm was evaluated.

**Results:**

Intra-class correlations ranged from 0.96 to 0.98. The correlations of XA-CCT_RA2,_ XA-CCT_RM2,_ XA-CCT_LA2,_ and XA-CCT_LM2_ with the corresponding CT-CCTs were 0.64, 0.65, 0.53, and 0.68, respectively. All CCTs were significantly prolonged in the vasospasm group (5.8–6.4 s) except for XA-CCT_LA2._ CT-CCT_A2_ of 5.62 was the optimal cut-off value for detecting vasospasm with a sensitivity of 84.2% and specificity 82.4%

**Conclusion:**

CT-CCTs can be used to interpret cerebral flow without deconvolution algorithms, and outperform both MTT and TTP in predicting vasospasm risk. This finding may help facilitate management of patients with SAH.

## Introduction

Endovascular treatment has been proven to be safe for patients with aneurysmal subarachnoid hemorrhage (SAH) [1]. However, despite successful management of ruptured aneurysms, vasospasm eventually develops and causes morbidity in 20–34% of patients [2, 3]. Therefore, early identification of patients at risk for vasospasm deserves attention so that timely treatment with angioplasty or intra-arterial administration of calcium channel blockers to improve neurologic outcomes can be considered [4, 5]. The incidence of vasospasm varies from 16–70% according to the criteria used: lowest when only clinical symptoms are considered and increasing when other criteria such as digital subtraction angiography (DSA) or transcranial Doppler are considered [6]. Delayed cerebral ischemia, defined as symptomatic vasospasm when confirmed by infarcted lesions on CT scans, effectively predicts mortality and severe disability at three months, but cannot be used in early stages to identify patients at risks [7].

An angiographic vasospasm is a newly developed luminal narrowing of intracranial vasculature in post-SAH control angiographies [6, 8]. Prolonged angiographic cerebral circulation time (CCT) upon admission is a risk factor for post-SAH vasospasm [8]. Angiographic CCT refers to the time difference between time to peak (TTP) for the cavernous ICA and the parietal vein, representing the time needed for blood to pass the brain parenchyma [9]. In addition to identifying vasospasm, quantitative angiographic CCT has proven to be robust in monitoring peritherapeutic intracranial hemodynamic changes in the endovascular treatment of stenotic-occlusive disease and carotid-cavernous fistula [10–14]. The major advantage of this application is its rapid processing within the angiosuite. Previously acquired standard DSA series can be used for analysis without extra radiation exposure and contrast medium administration.

CT perfusion (CTP) can detect cerebral perfusion deficits related to vasospasm. Decreased cerebral blood flow (CBF) and increased mean transit time (MTT) are the most useful indicators with a pooled sensitivity of 73%, and specificity of 93% [15]. Sanelli et al. showed that CTP on admission can predict the likelihood of subsequent vasospasm with sensitivity of 50–60%, and specificity of 70–91% [16]. Nevertheless, CTP is vulnerable to inter-observer variability at several points, including selection of ROIs, arterial input function and different de-convolution methods [17, 18]. In cases of acute stroke, perfusion mismatch deficits are remarkably decreased and thus accurate results can be automatically generated from various types of datasets [19]. In most cases of vasospasm, the disturbance of CCT occurs in small intracranial arteries, making the application of optimal cut-off values in CTP to differentiate vasospasm from control groups particularly challenging due to the subtle nature of the changes [3, 20].

Given reliable prediction of vasospasm by angiographic CCT (intra-arterial injection), we wondered whether the CCT derived from CTP (intra-venous injection) could serve as an immediate surrogate hemodynamic marker to predict future likelihood of vasospasm. Therefore, we conducted the current study to explore the relationship between CCTs defined by CTP and DSA, and to further test the feasibility of using different CCTs to predict subsequent SAH-related vasospasm.

## Materials and Methods

### Patient population

One-hundred and thirty–two patients with non-traumatic SAH were recruited. All patients received CT Angiography and CTP at the emergency service center. The average time interval between their visits and CTP was 2.8 hours, ranging from 2 to 6 hours. Forty-six cases with uncertain aneurysm locations received DSA for further evaluation. Forty-one cases with both diagnostic CTP and DSA images available and acquired within a 24-hour interval were enrolled in our study. All of these cases underwent surgical clippings and received control angiography or CT angiography. They were further divided into two groups: the vasospasm group was defined to include patients with clinical deterioration on the Glasgow Coma Scale (GCS) of more than 2 points, evidenced by newly developed luminal narrowing of intracranial vessels in either subsequent DSA or CTA, and after excluding other etiologies such as hydrocephalus, electrolyte imbalance or sedative overdose [3, 6, 8]. Remaining cases were included in the control group. *All cases in the control group also received CTP on admission*. The patients’ initial GCS scores, Fisher grades and follow-up CT scans were reviewed. Any infarcts on the CT scans correlated with the territory of the vasospasm were considered to be delayed cerebral ischemia. This study was approved by Institutional Review Board of Taipei Veterans General Hospital and all participants gave their written informed consent.

### DSA

**Fig 1:**
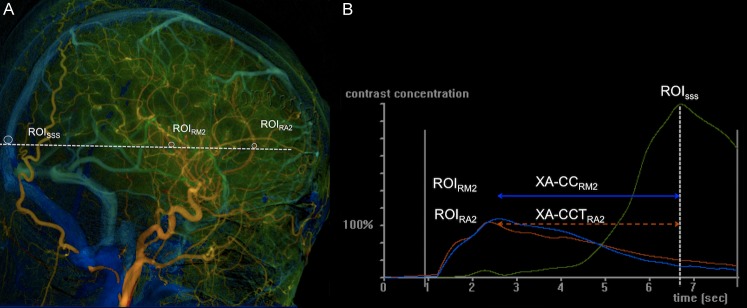
Lateral view of quantitative color-coded right common carotid DSA and the time density curves of ROIs. (A) Lateral view of quantitative color-coded right common carotid DSA. ROI_RA2_ was located at the greatest curvature of ACA. ROI_SSS_ and ROI_RM2_ were chosen by drawing an imaginary line (dashed line) parallel to the osteomeatal line to intersect SSS and the insular branch of the right-middle cerebral artery (RM2) respectively. (B) Time density curves of ROI_RA2_, ROI_RM2_, and ROI_SSS_. The dashed line intersecting the X-axis perpendicularly indicate the Tmax of ROI_SSS_. The XA-CCT_RA2_ (double-headed line) was defined as the time difference between the Tmax of ROI_RA2_ and that of ROI_SSS;_ the XA-CCT_RM2_ (dashed double headed line) was defined as the time difference between the Tmax of ROI_RM2_ and that of ROI_SSS._

All angiographies were performed in one flat-panel angiography machine (AXIOM-Artiszee^®^, Siemens Healthcare). A 4-French angiocatheter was placed at the level of the C4 vertebral body for the DSA. A fixed DSA imaging acquisition protocol, as previously reported by Lin et al [21] was used. Post-processing software (syngo *i*Flow^®^, Siemens Healthcare) was used to measure cerebral CCT based on time to peak (TTP) measurement with region of interest (ROI) placement on DSA [22]. The TTP for any selected ROI was defined as the time point at which the attenuation of the X-ray reached a maximum along the angiographic time frames. The reference time point, t = 0, was defined as the imaging time point of the selected DSA mask frame. A lateral view DSA was used for ROI selection to avoid confounding vasculature overlapping. On the right carotid artery DSA, a ROI was placed at the greatest curvature of the right ACA and named ROI_RA2_. An imaginary line parallel to the osteomeatal line was drawn to intersect the superior sagittal sinus (SSS) and the insular branch of the right-middle cerebral artery. Accordingly, the intersections were defined as ROI_sss_ and ROI_RM2,_ respectively (Fig 1A). The CCT measured on X-ray DSA (XA-CCT) was defined as the difference in TTP between the measured arterial ROI and the venous ROI. Therefore, two XA-CCTs (XA-CCT_RA2_ and XA-CCT_RM2_) were generated, based on the different arterial ROIs (ROI_RA2_ and ROI_M2_) selected in the ipsilateral DSA series (Fig 1B). Two additional XA-CCTs were generated (XA-CCT_LA2_ and XA-CCT_LM2_) from the left carotid artery DSA with the same measurement. ROI diameters were calculated so as to cover the calibers of the selected vessels without including imaging pixels outside the vessels [21]. One neuroradiologist and one angiographic technician with five years of experience in quantitative DSA, both unaware of the clinical conditions of the patients, analyzed all of the DSA datasets independently.

### CTP

**Fig 2:**
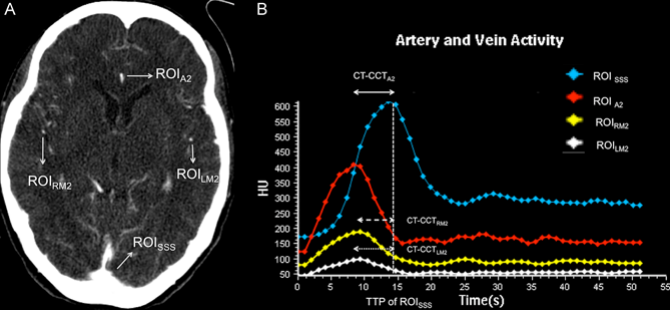
Axial view of the time-maximum intensity projection CTP and the time density curve of ROI. (A) An axial view of the time-maximum intensity projection CTP at the level where the ACA curved anteriorly and posteriorly. ROI_A2_ was chosen at the dominant ACA; ROI_RM2,_ ROI_LM2,_ and ROI_SSS_ were designated at the corresponding vessels. (B) Time density curve of ROI_A2,_ ROI_RM2,_ ROI_LM2,_ and ROI_SSS._ The dashed line intersected perpendicularly with the X-axis indicated the TTP of SSS. The CT-CCT_A2_ (double headed line) was defined as the time difference between the TTP of ROI_A2_ and that of ROI_SSS_; The CT-CCT_RM2_ (dashed double headed line) was defined as the time difference between the TTP of ROI_RM2_ and that of ROI_SSS_. The CT-CCT_LM2_ (dotted double headed line) was defined as the time difference between the TTP of ROI_LM2_ and that of ROI_SSS_.

CT angiography and CTP were performed in a multislice CT (Brillance^®^ CT, Philips Healthcare, Cleveland, USA). A total of 50 ml contrast (Ultravist^®^ 370; Bayer, Berlin, Germany) was injected via the right antecubital vein at a rate of 5 ml/s, followed by a 20 ml bolus flush of normal saline. The acquisition parameters were: 80 kVp, 100 mAs, and a 1 second fixed-scan interval that lasted 1 min. We positioned patients to ensure that the axial scans were parallel to the osteomeatal line during the CTP. The scan range started 2 cm above the tuberculum sellae to cover a 4 cm upward scanning range. The whole dataset was sent to a dedicated workstation (EBW^®^4.5 Philips Healthcare, Cleveland, USA) to generate cerebral blood volume (CBV), cerebral blood flow (CBF), mean transit time (MTT), and time to peak (TTP). Due to the spatial resolution on the CTP imaging, the observer located only the dominant ACA at the slice where it curved anteriorly–posteriorly. Two additional arterial ROIs located at the insular branches of the right and left-middle cerebral arteries at the same slice were selected, accordingly. The venous ROI was placed at the SSS on the same slice (Fig 2A). The time density curve (TDC) was generated after determining the arterial and venous ROIs (Fig 2B). Three CT circulation times (CT-CCT), namely, CT-CCT_A2,_ CT-CCT_RM2_ and CT-CCT_LM2,_ were generated based on the time difference between the TTP values of the selected arterial and venous ROIs mentioned above. One neuroradiologist with 10 years of experience with CTP and one CT technician with 7 years of experience, both unaware of the clinical conditions of the patients, independently analyzed all of the CTP datasets.

### Statistics

Intraclass correlation coefficients (ICC) for the measurements of the two observers were calculated for all Tmax, TTPs, CT-CCTs and XA-CCTs. Whenever there was a discrepancy, the average was used for further analysis. Pearson correlation coefficients between the three CT-CCTs, four XA-CCTs, and the GCS were calculated to verify the consistency of the CCTs. All CCTs and perfusion parameters for the vasospasm and control groups were compared using Student’s *t*-test. We used receiver operating characteristic curves (ROC) to evaluate the diagnostic performance of the statistically significant CCTs and perfusion parameters in predicting subsequent vasospasm. The maximum area under the curve of the ROC was used to determine optimal cutoff value. Significance was set to *p* < 0.05 for all statistical tests. Analyses were performed using SPSS^®^ 20 (IBM-SPSS, Chicago, USA).

## Results

The clinical symptoms and signs for all forty-one patients are summarized in Table 1. The mean time interval between the CTP and DSA scanning starts was 6.1 hours. The mean time interval between admission and vasospasm development was 7 ± 2.8 days. All of the treatments were smoothly. There were *not* significantly more subjects with higher Fisher grades in the vasospasm group than in the control group (*p* = 0.186). There was a significantly higher incidence of delayed cerebral ischemia in the vasospasm group (*p* <0.001). The ICCs for DSA-based TTP measurements between the two readers ranged from 0.92 to 0.98, and the ICCs for the CTP-based TTP measurements ranged from 0.85 to 0.99. The consistency of venous ROI placement and time measurement was not inferior to that of the arterial ROI in either the DSA or the CTP. Although there was less consistency for the DSA-based RA2 (ICC = 0.92) and CTP-based LM2 (ICC = 0.85) measurements, CCT measurement showed good consistency across the DSA- and CTP-based methods, with ICCs ranging from 0.96 to 0.98 (Table 2).

**Table 1:** Distribution of Patient Characteristics between control and vasospasm groups.

	Control	Vasospasm	p^†^
**n**	19	22	
**Sex (Male/Female)**	8/11	11/11	0.65
**Age**	54.5 ± 15.3	61.4 ± 14.0	0.14
**Initial GCS**	12.3 ± 3.9	10.3 ± 4.5	0.32
**Fisher Grade (I/II/III/IV)**	(6/6/1/6)	(5/2/2/13)	0.186
**Interval of two modalities**	5.8 ± 5.9	6.6 ± 5.4	0.72
**Interval between admission and vasospasm**	N/A	7 ± 2.8	N/A
**Delayed cerebral ischemia**	0	5	<0.001

GCS = Glasgow Coma Scale.

^†^ Mean comparisons were conducted using Student’s *t*-test for continuous variables and the Chi-square test for categorical variables.

**Table 2:** Intraclass-classification of Tmax in selected ROIs in DSA, TTP of selected ROIs in CTP, and calculated CCT.

**TTP of ROI in DSA**				
	**Read A**	**Reader B**	**ICC**	**Range**
**RA2**	3.3 ± 0.9	3.3 ± 0.9	0.92	0.82–0.96
**RM2**	3.5 ± 1.0	3.3 ± 1.0	0.96	0.92–0.98
**LA2**	3.1 ± 0.8	3.1 ± 0.7	0.97	0.97–0.99
**LM2**	3.2 ± 0.7	3.1 ± 0.7	0.98	0.96–0.99
**RSSS**	9.5 ± 2.5	9.4 ± 2.5	0.98	0.97–0.99
**LSSS**	9.2 ± 2.4	9.0 ± 2.4	0.95	0.88–0.97
**TTP of ROI in CTP**				
	**Read A**	**Reader B**	**ICC**	**Range**
**A2**	13.2 ± 4.7	13.2+4.1	0.99	0.99–0.99
**RM2**	14.3 ± 3.1	14.3 ± 4.0	0.91	0.81–0.96
**LM2**	13.7 ± 4.2	13.8 ± 4.0	0.85	0.81–0.95
**CCT**				
	**Read A**	**Reader B**	**ICC**	**Range**
**XA-CCT**_**RA2**_	5.7 ± 1.9	5.7 ± 1.8	0.97	0.94–0.99
**XA-CCT**_**RM2**_	5.9 ± 1.8	5.9 ± 1.9	0.97	0.94–0.99
**XA-CCT**_**LA2**_	6.0 ± 2.0	5.9 ± 1.9	0.96	0.91–0.98
**XA-CCT**_**LM2**_	5.9 ± 2.0	5.9 ± 2.0	0.97	0.94–0.98
**CT-CCT**_**A2**_	6.4 ± 2.1	6.4 ± 2.0	0.98	0.96–0.99
**CT-CCT**_**RM2**_	6.3 ± 2.0	6.3 ± 2.0	0.98	0.95–0.99
**CT-CCT**_**LM2**_	5.8 ± 2.0	5.9 ± 2.0	0.97	0.94–0.98

**Fig 3:**
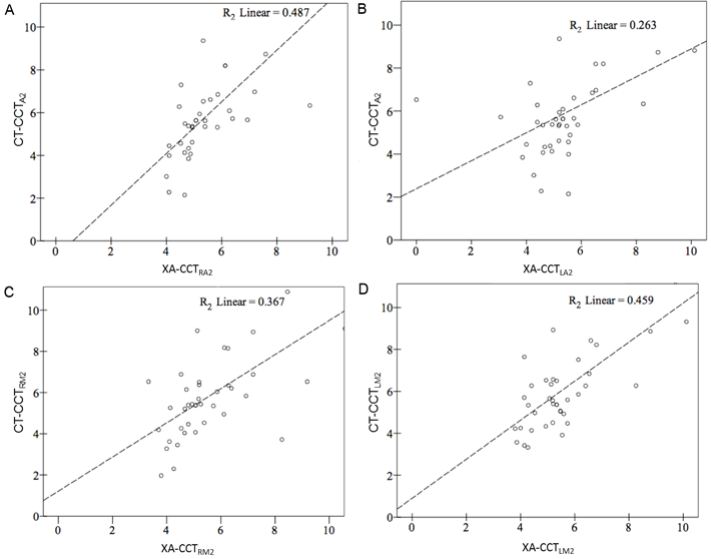
Correlation between cerebral circulation time defined by CT perfusion and cerebral circulation time defined by digital subtraction angiography. Correlation between (A) CT-CCT_A2_ and XA-CCT_RA2_; (B) CT-CCT_A2_ and XA-CCT_LA2_; (C) CT-CCT_RM2_ and XA-CCT_RM2_; and (D) CT-CCT_LM2_ and XA-CCT_LM2_. Y axis: CT-CCT, X axis: XA-CCT. CT-CCT: Cerebral circulation time defined by CT perfusion. XA-CCT: Cerebral circulation time defined by digital subtraction angiography. The subscripted abbreviations following CT-CCT and XA-CCT show different arterial ROIs used to calculate circulation time. RA2: second segment of right anterior cerebral artery; RM2: second segment of right-middle cerebral artery; LA2: second segment of left anterior cerebral artery; LM2: second segment of left-middle cerebral artery.

**Fig 4:**
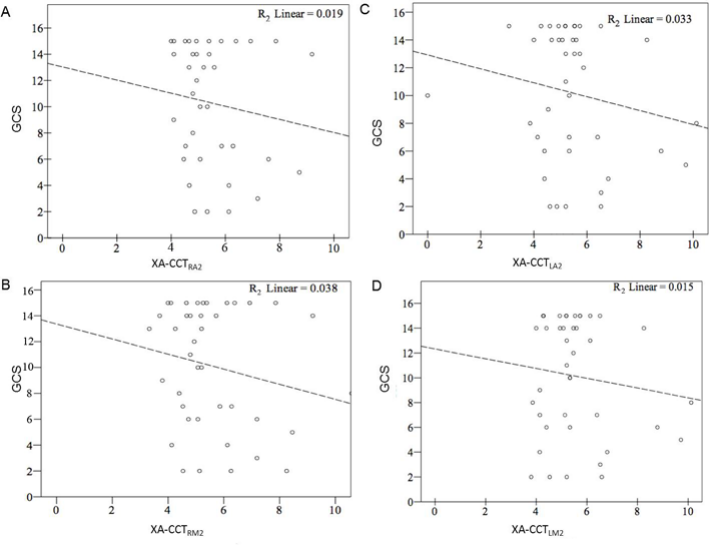
Correlation between GCS and four individual XA-CCTs. (A) XA-CCT_RA2,_ (B) XA-CCT_RM2,_ (C) XA-CCT_LA2,_ and (D) XA-CCT_LM2._ GCS: Glascow Coma Scale, XA-CCT: Cerebral circulation time defined by digital subtraction angiography. The subscripted abbreviations following XA-CCT show different arterial ROIs used to calculate circulation time. RA2: the second segment of the right anterior cerebral artery; RM2: insular branch of the right-middle cerebral artery; LA2: second segment of the left anterior cerebral artery; LM2: insular branch of left-middle cerebral artery.

**Fig 5:**
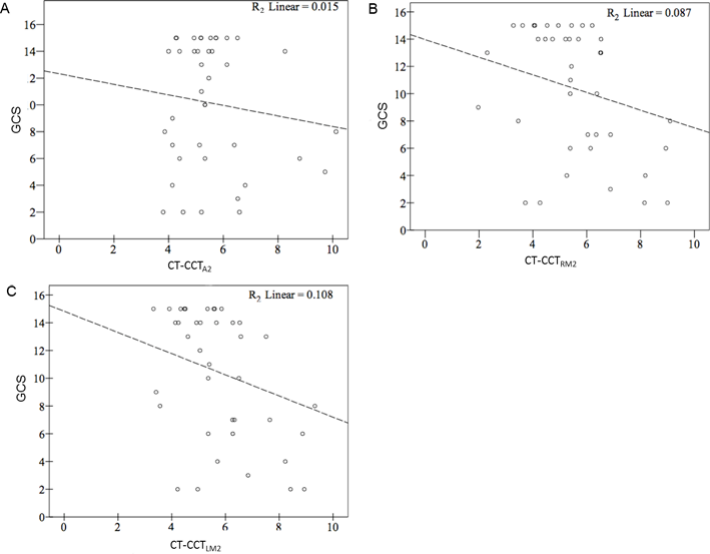
Correlation between GCS and thee individual CT-CCTs. (A) CT-CCT_A2,_ (B) CT-CCT_RM2,_ (C) CT-CCT_LM2._ GCS: Glascow Coma Scale, CT-CCT: Cerebral circulation time defined by CT perfusion. The subscripted abbreviations following CT-CCT show different arterial ROIs used to calculate circulation time. A2: second segment of the dominant anterior cerebral artery; RM2: insula branch of the right-middle cerebral artery; LM2: insular branch of the left-middle cerebral artery.

**Fig 6:**
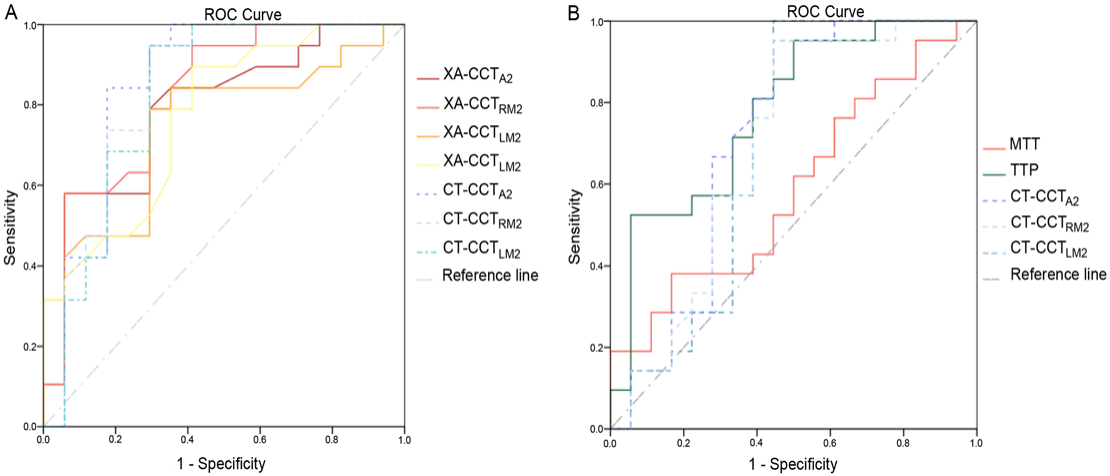
Receiver operating characteristic analysis. (A) Receiver operating characteristic analysis of all XA-CCTs and CT-CCTs. (B) Receiver operation characteristics analysis of all CT-CCTs, MTT and TTP

High and significant correlations were found between XA-CCT and CT-CCT based on the current ROI placements and time measurements (R^2^ ranged from 0.263 to 0.487) (Fig 3). The correlation coefficients were lowest for LA2 (r = 0.53), followed by LM2 (r = 0.62), RA2 (r = 0.64) and RM2 (r = 0.65), as arterial ROIs. None of the correlation for GCS and XA-CCT were significant (R^2^ ranged from 0.015 to -0.108 (Fig 4). Only CT-CCT_LM2_ showed a mild correlation with GCS (R^2^ = 0.108). Neither CT-CCT_RM2_ nor CT-CCT_A2_ were significantly correlated with GCS (Fig 5). Apart from XA-CCT_LA2_, the remaining angiographic and CT CCTs in the vasospasm group were significantly prolonged compared to those in the control group. For CTP, only MTT and TTP were significantly prolonged in the vasospasm group compared to the control group (Table 3). The ROC curves of XA-CCT, CT-CCT, MTT and TTP are provided in Fig 6. All CT-CCT measures displayed superior diagnostic accuracy compared to the XA-CCTs, and were followed by TTP and MTT in differentiating the vasospasm from control groups (Table 4).

**Table 3:** Different cerebral circulation times, and perfusion parameters between vasospasm and control groups.

**Circulation Time**	**Control (N = 19)**	**Vasospasm (N = 22)**	***P***
**XA-CCT**_**RA2**_	4.9 ± 0.9	6.0 ± 1.3	0.08*
**XA-CCT**_**LA2**_	4.6+1.0	5.8+2.4	0.055
**XA-CCT**_**RM2**_	4.7 ± 1.3	5.8 ± 2.1	0.045*
**XA-CCT**_**LM2**_	4.9 ± 0.8	5.9 ± 2.1	0.05*
**CT-CCT**_**A2**_	5.0 ± 2.6	6.4 ± 2.2	0.03*
**CT-CCT**_**RM2**_	4.9+2.2	6.4+2.2	0.03*
**CT-CCT**_**LM2**_	5.2 ± 2.1	6.7 ± 1.7	0.01*
**Perfusion**	**Control (N = 19)**	**Vasospasm (N = 22)**	***P***
**CBV**	4.0 ± 1.6	3.3 ± 1.0	0.30
**CBF**	30.0 ± 21.0	27.2 ± 17.4	0.35
**MTT**	5.5 ± 1.1	6.3 ± 2.1	0.02*
**TTP**	15.1. ± 7.1	17.9 ± 3.1	0.02*

* Student’s *t*-test p-value was less than 0.05.

**Table 4:** Optimal Cutoff Values of Ten all CCT, MTT, and TTP for Predicting Vasospasm.

ROI-variable	AUC	*P*	Optimal cutoff value	Sensitivity	Specificity
**XA-CCT**_**RA2**_	0.788(0.636–0.940)	0.003*	5.01	78.9%	70.6%
**XA-CCT**_**LA2**_	0.746(0.582–0.910)	0.001*	5.19	78.9%	70.6%
**XA-CCT**_**RM2**_	0.824(0.684–0.963)	0.012*	4.70	94.7%	58.8%
**XA-CCT**_**LM2**_	0.769(0.615–0.923)	0.006*	5.10	89.5%	58.8%
**CT-CCT**_**A2**_	0.851(0.710–0.993)	0.000*	5.62	84.2%	82.4%
**CT-CCT**_**RM2**_	0.837(0.694–0.981)	0.001*	5.32	94.7%	70.6%
**CT-CCT**_**LM2**_	0.824(0.674–0.974)	0.001*	5.35	94.7%	64.7%
**CBV**	0.623(0.552–0.695)	0.001*	2.74	41.3%	80.6%
**CBF**	0.627(0.555–0.698)	0.001*	34.35	65.9%	60.2%
**MTT**	0.590(0.410–0.770)	0.001*	6.13	33.3%	83.3%
**TTP**	0.778(0.630–0.925)	0.001*	17.3	52.2%	94.0%

*Student’s *t*-test p-value was less than 0.05.

## Discussion

Although manual selection of ROIs is still needed in current versions of TDC analysis for both quantitative color-coded DSA and CTP, the consistency of inter-observer TTP ratings and calculated CCTs were reasonably high. In contrast to previous reports, we did not find inferior consistency in venous ROIs compared to arterial ROIs [21]. The cerebrovascular reserve and integrity of the circle of Willis might vary and change the CCT measurements [23–25]. The better correlations of measured AX-CCT and CT-CCT using ROI_RA2_ instead of ROI_LA2_ are likely due to the higher incidence of hypoplasia of A1 in our study. The competing flows in the unilateral DSA series from the dominant (right) ACA via the anterior communicating artery would change the TDC waveform and subsequently decrease the accuracy of Tmax in ROI_RA2_ [21]. Although both used TDC to evaluate cerebral hemodynamics, the CTP was performed with intravenous administration of a bolus of contrast, whereas the DSA was administered via intra-arterial injection. Compared to intravenous injection, intra-arterial injection alters the waveform of the blood flow because of the power injector and thus does not perfectly represent physiologic flow. Nevertheless previous studies have shown that XA-CCT provides reliable hemodynamic monitoring [8–10]. In the current study, we found a high correlation between TDC with CTP (intra-venous injection) and DSA (intra-arterial injection), further supporting the feasibility of using CT-CCT as a surrogate to evaluate cerebral perfusion in SAH patients. This parameter is immediately available and free of variation from different perfusion software, and serves as an adjunct to CBF, MTT, TTP across different platforms [15, 26].

According to Sanelli et al, patients with lowered CBF, prolonged MTT and reduced CBF in CTP upon admission are predisposed to develop vasospasm later on. CBV and CBF have higher specificity (89–91%), but their sensitivities are low (36–50%). MTT has moderate specificity (70%) and sensitivity (61%). In contrast to previous studies, we did not find CBF to be a useful predictor of subsequent vasospasm [16]. Both MTT and TTP showed low sensitivity with high specificity in detecting vasospasm. A plausible explanation is that all CCTs, as well as TTP, were direct time-dependent variables and therefore were more sensitive to intravascular flow changes in response to constrictions of vessel walls in the early stage of vasospasm. MTT was calculated from deconvolution or the central volume principle to reflect the duration required for blood to go through the brain parenchyma, and thus was a mixed measurement of blood flow and “resistance” of the brain tissue [27]. The influence of different intervals in CCT and angiography was not validated in the current study. Given the internal interpolation algorithm used for TDC, we speculate that the influence of the acquisition interval is limited, but further study is needed for validation [28].

According to Krayenbűhl et al [9, 29], SAH patients with a circulation time of more than 5.5 s are considered to have developed a vasospasm. In our study, CCTs in the vasospasm group ranged from 5.9 to 6.5 s. Longer CCTs in our study resulted from our choice of using A2 and M2 as the arterial ROIs, and SSS as the venous ROI. The distance for the blood to travel was longer, compared to the distance from the cavernous portion of the internal carotid artery to the parietal vein in Krayenb**ű**hl et al [29]. Iseda et al found a high correlation between CCT and SPECT, and their mean CCTs for the SAH patients with vasospasm was 4.1 seconds[13]. The shorter CCTs likely resulted from the shorter distance between the two reference points: the middle cerebral artery and cortical artery.

The low correlation between level of consciousness and XA-CCT or CT-CCT is probably due to several factors, including comorbid hydrocephalus, cerebral edema and fluctuations in the patients’ levels of consciousness between two examinations in the acute stage. The original Fisher grading system and its modified versions uses the distribution of SAH and IVH to evaluate the risk that patients will suffer delayed ischemic insult [30]. By using CCT measurement, the initial vascular response was quantified with a hemodynamic index. This index not only served as a predictor but also an indicator of vasospasm treatment results during the series follow-up [31].

Our study has several limitations. First, the study was retrospective, and the patient population was fairly small and limited to aneurysmal SAH patients with both CTP and DSA available. Therefore, potential lead-time bias and recall bias cannot be excluded. Second, the CTP coverage was smaller than that for DSA, thereby making it impossible to register all corresponding ROIs from the DSA in the CTP. Further studies using whole brain perfusion might optimize the comparison [32]. The overlapping anatomical structure in the two-dimensional DSA renders it impossible to correlate perfusion parameters between CTP and, for example, CBF with XA-CCT. Third, our study only revealed that intra-arterial and intra-venous injection protocols produced similar TDCs, but the interaction of heart rate and blood pressure on TDC was not made clear. Further studies using different FWHM time parameters, for example, maximum slopes, to elucidate mathematic relationships can improve the reliability of CBV by intra-arterial administration [33].

Struffert et al used dynamic scanning by flat-panel detectors to generate perfusion imaging and successfully detect perfusion deficits in acute stroke patients within the angiosuite [34]. This method can potentially monitor flow changes while treating patients with vasospasm by angioplasty or intra-arterial calcium channel blocker infusion. Nevertheless, compared to XA-CCA, this application required extra contrast injection for hemodynamic assessment. We confirmed that the TDC curves were similar for CTP and DSA, suggesting that intra-arterial protocols can successfully produce diagnostic images and can reduce contrast by 50% as well as reducing subsequent contrast-induced nephrotoxicity[35].

## Conclusion

The similarity in TDCs between DSA and CTP can potentially help establish hemodynamic models with intra-arterial injections for flat-panel detector perfusion imaging. Like XA-CCT, CT-CCTs can be used in interpret cerebral flow without deconvolution algorithms, and outperform both both MTT and TTP in predicting vasospasm risk. This finding may help facilitate management of patients with SAH in the future when an appropriate prospectively study is performed that adequately addresses the relative risk and predictive values of these studies and algorithms for symptomatic vasospasm and delayed ischemic neurologic deficits.
